# Pro-Inflammatory Properties of Salivary Gland-Derived Fibroblasts—Implications in Sjögren’s Disease

**DOI:** 10.3390/cells14080558

**Published:** 2025-04-08

**Authors:** Matthias Brunner, Daniel Guggisberg, Marco Sprecher, Ondrej Pastva, Kristina Bürki, Miranda Houtman, Marco Kreuzer, Sara Andrea Krättli, Laura Jahnke, Mila Roceri, Rémy Bruggmann, Muriel Elhai, Britta Maurer, Thomas M. Marti, Caroline Ospelt, Kerstin Klein

**Affiliations:** 1 Department of Rheumatology and Immunology, Inselspital, Bern University Hospital, University of Bern, 3008 Bern, Switzerland; 2 Lung Precision Medicine (LPM), Department for BioMedical Research, 3008 Bern, Switzerland; 3 Department of Rheumatology, University Hospital Zurich, University of Zurich, 8091 Zurich, Switzerland; 4 Interfaculty Bioinformatics Unit, University of Bern, 3012 Bern, Switzerland; 5 Department of General Thoracic Surgery, Inselspital, Bern University Hospital, 3008 Bern, Switzerland

**Keywords:** Sjögren’s disease, salivary gland, inflammation, fibroblast, enhancer, bromodomain

## Abstract

Salivary gland dysfunction is a hallmark of Sjögren’s disease (SjD). Here, we investigated the pro-inflammatory properties of salivary gland-derived fibroblasts (SGF) that were cultured from minor salivary gland (MSG) tissues of patients with SjD and controls. SGF from patients with SjD exhibited higher rates of proliferation compared to controls. RNA sequencing revealed pronounced pro-inflammatory properties of SGF in response to stimulation with IL1 and polyI:C, with an activation of “interferon responses”, “JAK STAT”, and “NF-kappa B” signaling, as well as ”complement” pathways. In addition to encoding pro-inflammatory transcripts, stimulated SGF featured increased expression of a number of non-coding enhancer RNAs (eRNAs) that we originally identified in TNF-stimulated synovial fibroblasts (FLS) by CAGE sequencing. We confirmed the expression of selected eRNAs in SGF and FLS through time-course experiments upon stimulation with different pro-inflammatory stimuli using real-time PCR. Furthermore, we detected eRNAs for IL6 (eIL6) and IL8 (eIL8#3) in MSG tissues. Treatment of SGF with the bromodomain inhibitor I-BET suppressed IL1- and LPS-induced expression of all eRNAs tested, as well as their associated pro-inflammatory coding transcripts. Transfection of SGF with antisense nucleotides targeting eCCL20 reduced the LPS-induced expression of this eRNA, as well as CCL20 expression and secretion. Together, our data highlight similarities between SGF and FLS regarding their activation under inflammatory conditions.

## 1. Introduction

Sjögren’s disease (SjD; formerly called Sjögren’s syndrome) is a chronic autoimmune disease characterized by dysfunction of exocrine glands, particularly salivary and lacrimal glands, resulting in manifestations such as dry mouth and dry eyes. Systemic organ involvement occurs in 30–40% of patients [1]. While SjD can manifest as a primary disease, it is frequently seen in conjunction with other autoimmune disorders, such as rheumatoid arthritis (RA), systemic lupus erythematosus (SLE), or systemic sclerosis (SSc) [2]. Treatment for SjD remains largely symptomatic, focusing on alleviating glandular dryness and managing systemic manifestations.

The pathogenesis of SjD involves a complex interplay between genetic risk factors and environmental triggers, such as viral infections [3]. Together, they activate salivary gland epithelial cells (SGEC), with a subsequent activation of and interplay with cells of the innate and adaptive immune system, leading to glandular damage and functional impairment [4]. Whereas the role of fibroblasts (FLS) in RA is well established [5,6], salivary gland-derived fibroblasts (SGF) and their role in SjD are only beginning to be explored [7,8,9,10]. Fibroblasts, the key cellular players in tissue repair and extracellular matrix (ECM) production, are integral for maintaining tissue architecture in a healthy tissue environment. In chronic inflammation, fibroblasts get activated, perpetuate ongoing inflammation, and promote the switch from an acute to a chronic disease [5,10,11,12]. Single cell RNA-sequencing of synovium, salivary gland, lung, and intestine discovered the existence of shared types of fibroblasts in chronically inflamed tissues that interact with immune and vascular cells [10]. In addition, fibroblasts activation was shown to be a key event in the formation of tertiary lymphoid structures (TLS) in salivary gland tissues [7,8,9]. Given that fibroblast activation precedes lymphocyte infiltration into salivary gland tissues, fibroblasts may play an early and pivotal role in chronic salivary gland inflammation.

There is growing evidence that epigenetic mechanisms underlie cell activation in chronic inflammation. Cell-type- and stimulus-specific enhancers have been recognized as key DNA-regulatory elements in autoimmune diseases, that can get globally activated and act as intermediaries in linking environmental and genetic risk factors to an altered gene expression [13]. Approximately 30–50% of enhancers are transcribed bidirectionally into a class of non-coding RNAs termed enhancer RNAs (eRNAs), a hallmark of active enhancers [14]. Aberrant regulation of super-enhancer-associated eRNAs has been linked to cancer development and immune-mediated diseases [15].

In this study, we aimed to culture and characterize the inflammatory response of human primary SGF derived from minor salivary gland (MSG) tissues to provide, in addition to SGEC, an in vitro model for studying some aspects of SjD. We provide here a comprehensive transcriptome data set of stimulated SGF, mimicking a pro-inflammatory milieu with using interleukin 1 (IL1) and poly(I:C) (pIC). Given the similarities previously identified between SGF and RA FLS in chronically inflamed tissues [10], we compared the activation of transcribed enhancers, eRNAs, in these two cell types.

## 2. Materials and Methods

### 2.1. Patient Samples and Cell Preparation

**Figure 1:**
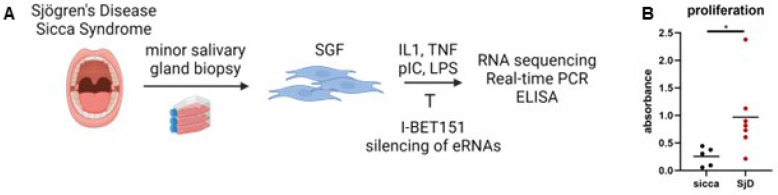
SGF in SjD. (**A**) Project outline (**B**) SGF proliferation assessed by BrdU assay. SGF from patients with sicca syndrome are shown as black dots, and SGF from patients with confirmed SjD as red dots. * *p* < 0.05.

We obtained MSG tissues from patients with SjD or patients with sicca symptoms who did not meet the classification criteria [16] at the University Hospital Zurich, Switzerland. To investigate the role of stromal fibroblasts in SjD, we established cultures of primary SGF from MSG biopsies of patients with suspected SjD and sicca symptoms (Figure 1A). Patients were afterwards classified as definite SjD based on the most recent classification criteria [16]. Patients not matching the classification criteria served as the control group and are labelled as “sicca”. Five patients with SjD had at least one additional autoimmune disease: two of them having thyroiditis, one having RA, one having SSc, one having SLE, and one having celiac disease. Nine patients with SjD had at least one extraglandular manifestation (Table 1). Two patients in the sicca control group had an additional undifferentiated collagenosis. MSG tissues were minced with a scalpel into small pieces, followed by one hour of enzymatic digestion in freshly prepared dispase II (Merck & Cie, Buchs, Switzerland) solution (75 mg of dispase [1–2 U/mg] in 50 mL D-PBS) on a magnetic stirrer at 37 °C. The cell suspension was filtered through a 0.22 µM cell strainer. Cells were pelleted by centrifugation and cultured in six-well plates in DMEM/F12 medium (Life Technologies, Zug, Switzerland; 11320074) containing 10% heat-inactivated fetal bovine serum, 2.22 mM L-glutamine, 0.0089 M HEPES (pH 7.4), 0.444 µg/mL amphotericin B, and 44.4 IU/mL penicillin-streptomycin (all Gibco). Cells were used between passages four to ten.

We obtained synovial tissues from hand, shoulder, and knee joints of patients with RA undergoing joint replacement surgery at the Schulthess Clinic Zurich, Switzerland. All patients fulfilled the criteria for the classification of RA [17]. FLS were isolated and cultured as described elsewhere [18] and used between passages four and eight for all experiments.

Lung tissues were obtained from the unaffected (healthy) surrounding human lung tissue during tumor resections at the University Hospital Bern. Lung fibroblasts (LF) were cultured as described above for SGF. All patients provided informed consent prior to inclusion in the study. The study was approved by the ethics committees of the Cantons of Zurich (approval numbers 2019-00115 and 2019-00674) and Bern (approval numbers 2019-02411 and 2018-01801), Switzerland. Clinical characteristics and treatment of patients with and without SjD are listed in Table 1. Clinical characteristics of patients with RA are listed in Supplementary Table S1.

### 2.2. FACS Analysis

SGF were harvested, washed with PBS containing 1% bovine serum albumin, and stained with APC anti-CD90 (BD Biosciences, Allschwil, Switzerland), FITC anti-CD326/Epcam (Miltenyi Biotec, Adliswil, Switzerland), eFluor450 anti-CD45 (Life Technologies, Zug, Switzerland), and APC Cy7 anti-Podoplanin (Biolegend, Amsterdam, The Netherlands), as well as their specific IgG Isotype control. All stainings were conducted as single stains. Flow cytometry analysis was performed using the Aurora 5-Laser spectrum analzyer (Cytek Biosciences, Amsterdam, The Netherlands) and visualized using Flowjo 10.10.0.

### 2.3. Proliferation Assay

1 × 10^4^ cells were seeded in 96-well plates. SGF proliferation was measured by BrdU assay (Merck & Cie, Buchs, Switzerland) after 24 h.

### 2.4. Treatment of Cells

SGF, FLS, and LF were stimulated with TNF (1, 3, 6, and 24 h; 10 ng/mL; Bio-Techne, Zug, Switzerland), IL1 (1, 24 h; 1 ng/mL; Life Technologies, Zug, Switzerland), or the Toll-like receptor agonists poly(I:C) (pIC; 1, 24 h; 10 μg/mL; InvivoGen, Tolouse, France) and LPS (1, 24 h; 100 ng/mL; InvivoGen, Tolouse, France). Where indicated, SGF were treated with the BET bromodomain (BRD) protein inhibitor I-BET151 (1 µM; 24 h; Bio-Techne, Zug, Switzerland) or matched amounts of DMSO (controls; Merck & Cie, Buchs, Switzerland). IL1 or LPS were either added simultaneously (24 h) or 1 h prior to harvesting the cells.

### 2.5. RNA Sequencing of SGF

SGF from patients with SjD (*n* = 3) were stimulated with IL1 and pIC, respectively, for 24 h. All three patients were positive for anti-SSA/Ro auto-antibodies, had a focus score of at least 1, and fulfilled the classification criteria for SjD [16]. Total RNA from SGF was isolated using the RNeasy Mini Kit (Qiagen, Zug, Switzerland) and quality was evaluated by Bioanalyzer measurements (Agilent Technologies, Basel, Switzerland). Library preparation and RNA sequencing (RNA-seq) were performed at the Next Generation Sequencing (NGS) Platform, University of Bern. Libraries for RNA-seq were generated using the Illumina Stranded mRNA-standard (LIBR03) kit. The quality and quantity of the generated libraries were tested by Bioanalyzer measurements. Libraries were sequenced using Illumina NovaSeq 6000 (Illumina, San Diego, CA, USA), with paired-read approaches. Quality control (QC) was conducted using FASTQC (Version 0.12.0). High-quality reads were mapped to the human reference genome (hg19) using HiSat2. Counts per gene were assessed using FeatureCounts. Differentially expressed genes (DEGs) between unstimulated and stimulated SGF were analyzed using DESeq2. Gene ontology overrepresentation analysis for biological processes of DEGs (log2 fold change 1.0, padj < 0.05) and pathway enrichment analysis (Molecular Signatures Database; MSigDB; padj < 0.05) were performed using topGo and clusterProfiler R Bioconductor packages.

### 2.6. ELISA

Cell culture supernatants of SGF were collected 24 h after stimulation. IL6, IL8, BAFF, CCL20, CCL2, CCL5, CXCL10, and CXCL1 were measured using DuoSet ELISA kits (R&D systems).

### 2.7. Cap Analysis of Gene Expression Followed by Sequencing (CAGE-Seq)

FLS (*n* = 9) were stimulated with TNF (10 ng/mL) or left untreated for 24 h. CAGE-seq was performed previously [19], and data sets are available in the GEO repository accession GSE163548 (https://www.ncbi.nlm.nih.gov/geo/query/acc.cgi?acc=GSE163548, accessed on 1 April 2025). The interaction of bidirectionally transcribed enhancers (eRNAs) and coding transcripts was predicted by co-expression analysis. The sequences of eRNAs were extracted from CAGE-seq data to design primers for real-time PCR.

### 2.8. Real-Time PCR

Total RNA was isolated using the RNeasy Mini Kit (Qiagen) with on-column DNA digestion. RNA was reversed-transcribed as previously described [18]. Real-time PCR (7900HT Real-Time PCR system, Life Technologies, Zug, Switzerland) was performed using self-designed primers (Microsynth, Balgach, Switzerland; Supplementary Table S2) and SYBR green (Roche: Cologne, Germany). Dissociation curves and samples containing the untranscribed RNA were measured in parallel. Constitutively expressed human ribosomal protein large P0 (RPLP0) was measured for internal standard sample normalization, and relative mRNA expression levels were calculated using the comparative threshold cycle method (ΔΔCt) [20].

### 2.9. Silencing of eRNAs in SGF

1.5 × 10^5^ SGF were transfected with antisense LNA gapmeRs (10 nM) targeting eIL8#3 and eCCL20, or gapmeR control (Qiagen, Zug, Switzerland), using lipofectamine (Life Technologies, Zug, Switzerland). 24 h after transfection, cells were stimulated with LPS (100 ng/mL) for 1 h and 24 h, respectively. Cells were harvested for RNA isolation and supernatants were collected for ELISA.

### 2.10. Statistical Analysis

GraphPad Prism (10.0.2) software was used for the statistical analysis of experimental data sets. Individual data points in all experiments represent biological samples from different patients. Data were tested for normal distribution using the Shapiro-Wilk test. Differences between experimental groups with normally distributed data were analyzed by analysis of variance (ANOVA) followed by Tukey’s multiple comparison test. For non-normally distributed data, the Friedmann test followed by the post hoc Dunn’s multiple comparison test was used. Data are reported as means ± standard deviations. *p* values < 0.05 were considered significant.

## 3. Results

### 3.1. Fibroblasts from Patients with Sjögren’s Disease Exhibit Increased Proliferation

SGF from patients with SjD exhibited increased proliferation compared to SGF from controls (Figure 1B). FACS analysis indicated that SGF were positive for the stromal markers CD90 and PDPN but negative for leukocyte and epithelial cell markers CD45 and Epcam, respectively (Supplementary Figure S1).

### 3.2. Salivary Gland Fibroblasts Respond to Pro-Inflammatory Stimuli

**Figure 2:**
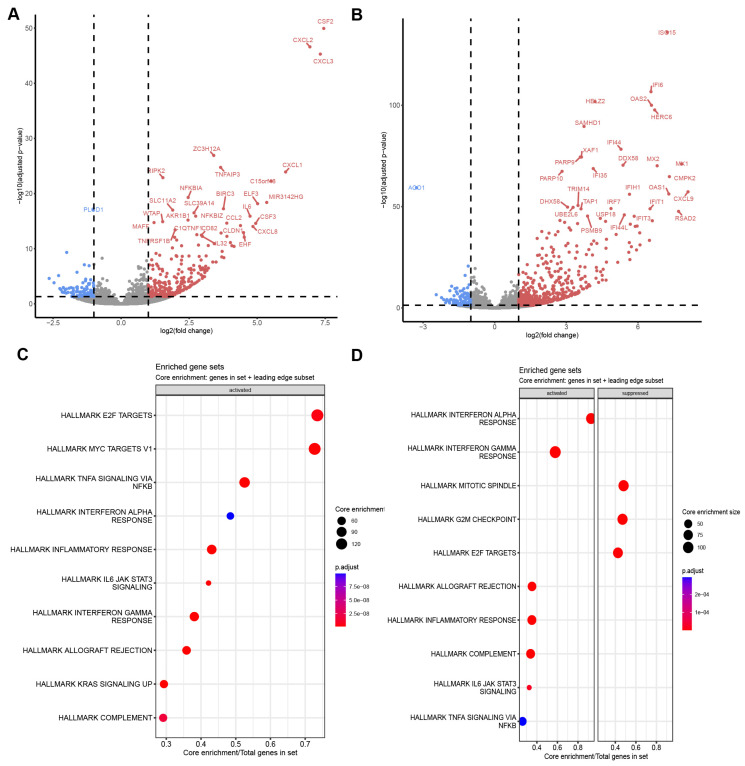
RNA-seq of SGF. SGF from patients with SjD were stimulated with (**A**) IL1 or (**B**) pIC for 24 h. Changes in gene expression were analyzed by RNA-seq. DEG (log2 FC > 1; padj < 0.05) are shown in Volcano plots. Dot plots of enriched gene sets (MSigDB) for (**C**) IL1 and (**D**) for pIC-stimulated SGF are shown.

To investigate the inflammatory response of SGF, we stimulated them with IL1 and pIC, respectively, and analyzed their response by RNA-seq. IL1 stimulation induced 253 genes and suppressed 91 genes in SGF (log2 FC >1; padj < 0.05; Figure 2A). DEG in IL1-stimulated SGF were enriched in biological processes (GO BP) and Hallmark pathways (MSigDB) associated with “chemotaxis”, “leukocyte activation and migration”, “NF-kappa B signaling”, “JAK STAT signaling”, “interferon signaling”, “KRAS signaling”, and “complement” (Figure 2C; Supplementary Table S3). Stimulation of SGF with pIC induced 577 genes and suppressed 114 genes (log2 FC >1; padj < 0.05; Figure 2B), with a profound activation of “interferon responses”, followed by “complement”, “JAK STAT signaling”, and “NF-kappa B signaling” (Figure 2D; Supplementary Table S4). In contrast to IL1-stimulated SGF, where we did not detect suppressed pathways, “mitotic spindle”, “G2M checkpoint”, and “E2F targets” were suppressed in pIC-stimulated SGF.

**Figure 3:**
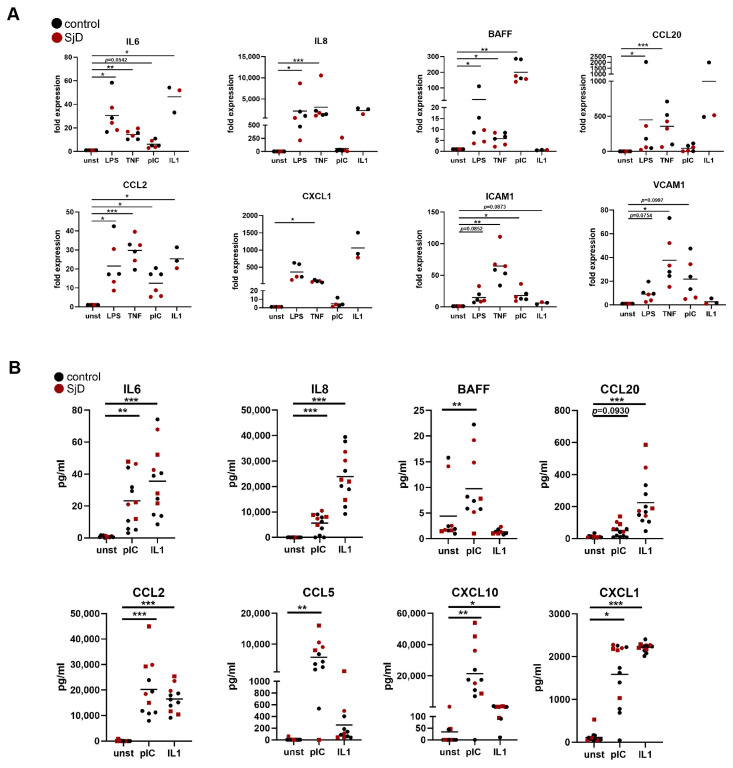
Pro-inflammatory response of SGF. SGF were treated with LPS, TNF, pIC, or IL1 for 24 h. (**A**) Changes in gene expression were measured by real-time PCR. (**B**) Secretion of cytokines and chemokines was measured by ELISA. * *p* < 0.05, ** *p* < 0.01, *** *p* < 0.005.

To further investigate the pro-inflammatory response of SGF, we analyzed the expression of selected target genes upon stimulation with different pro-inflammatory cytokines (TNF, IL1) and Toll-like receptor ligands (pIC, LPS). SGF responded to all different stimuli and induced the expression of cytokines (IL6, IL8), chemokines (CCL20, CCL2, CXCL1, BAFF), and adhesion molecules (ICAM1, VCAM1) (Figure 3A). Furthermore, SGF secreted the cytokines and chemokines IL6, IL8, CCL20, CCL2, CCL5, CXCL10, and CXCL1 upon stimulation with pIC or IL1 into cell culture supernatants (Figure 3B). Levels of induction were similar in SGF derived from patients with SjD and control SGF for all targets measured, with huge interpatient variability.

### 3.3. Pro-Inflammatory Stimuli Induce Shared Enhancers in Different Types of Fibroblasts

**Figure 4:**
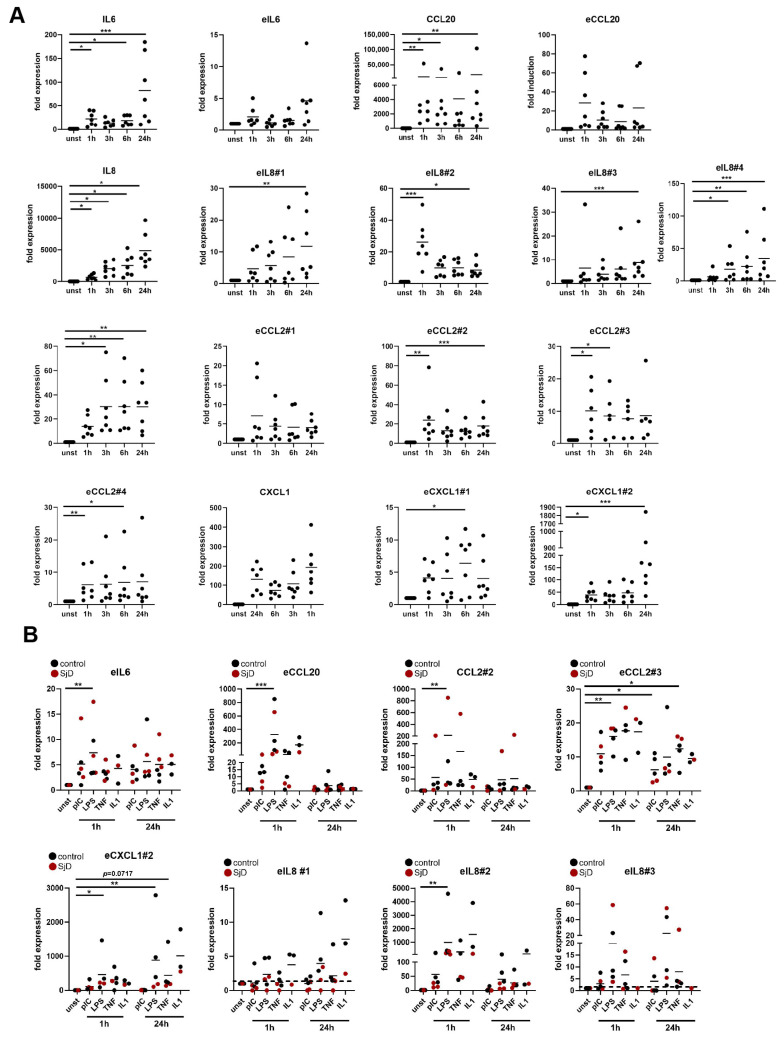
Pro-inflammatory stimuli induce the expression of eRNAs. (**A**) FLS were stimulated with TNF for 1, 3, 6, and 24 h. The expression of eRNAs and their corresponding coding genes was measured by real-time PCR. (**B**) SGF were treated with pIC, LPS, TNF, or IL1 for 1 h and 24 h. The expression of eRNAs was measured by real-time PCR. * *p* < 0.05, ** *p* < 0.01, *** *p* < 0.005.

To study the regulation of the pro-inflammatory response of SGF, we compared their enhancer activation upon stimulation to those of RA FLS and LF. We have previously mapped the enhancer landscape of RA FLS and identified TNF-induced, bidirectionally transcribed eRNAs using CAGE-seq [18,19]. We selected four eRNAs for *IL8* and *CCL2*, respectively, one for *CCL20* and two for *CXCL1* for detailed further analysis. The enhancer/promoter region of *IL6* was selected based on previous studies indicating a profound role of a regulatory region around −500 bp upstream of the *IL6* transcription start site (TSS) that orchestrates IL6 expression in FLS in a cell type-specific manner [21,22]. The selected enhancers were located upstream (*eCCL2*#1, *eCCL2*#2, *eCXCL1*#2, *eIL6*#1, *eIL8*#1-3), downstream (*eCCL2*#3, *eCCL2*#4, *eCXCL1*#1), and intronic (*eCCL20*) at distances between 300 bp to 35.6 kb relative to the TSS of the respective coding genes (Table 2). Their interaction with the coding gene was predicted based on the co-expression of the eRNA and the coding transcript. We extracted the sequences of eRNAs from RA FLS from CAGE-seq data sets (Supplementary Figure S2A,B) and measured their time-dependent induction upon TNF stimulation by real-time PCR (Figure 4A). Time-course experiments in RA FLS revealed the existence of different patterns of eRNAs: (a) eRNAs, that peaked at 1 h (eCCL20, eIL8#2, eCCL2#1), (b) at 6 h (eCXCL1#1), (c) or at 24 h (eIL6, eIL8#1, eIL8#3, eIL8#4, eCXCL1#2) after stimulation, and (d) eRNAs that were stably expressed over the time points (eCCL2#2, #3, #4). These eRNAs were not only induced by TNF but also by stimulation of FLS with IL1, pIC, and LPS (Supplementary Figure S3A).

Similar to the eRNA expression in FLS, we detected eRNAs for IL6, CCL20, CXCL1, IL8, and CCL2 in SGF and LF, with all pro-inflammatory stimuli inducing their expression (Figure 4B, Supplementary Figure S3B). We restricted our analysis to eRNAs with the highest expression. Together, the data indicate that inflammatory gene enhancers are shared in fibroblasts from different localization and diseases. However, we detected some differences regarding the peak of eRNA expression (eCCL2#2, eCCL2#3) and the potency of different stimuli to induce individual eRNAs (eIL8#2, eCXCL1#2).

### 3.4. The Expression of eRNAs Is Detectable in Salivary Gland Tissue Samples

**Figure 5:**
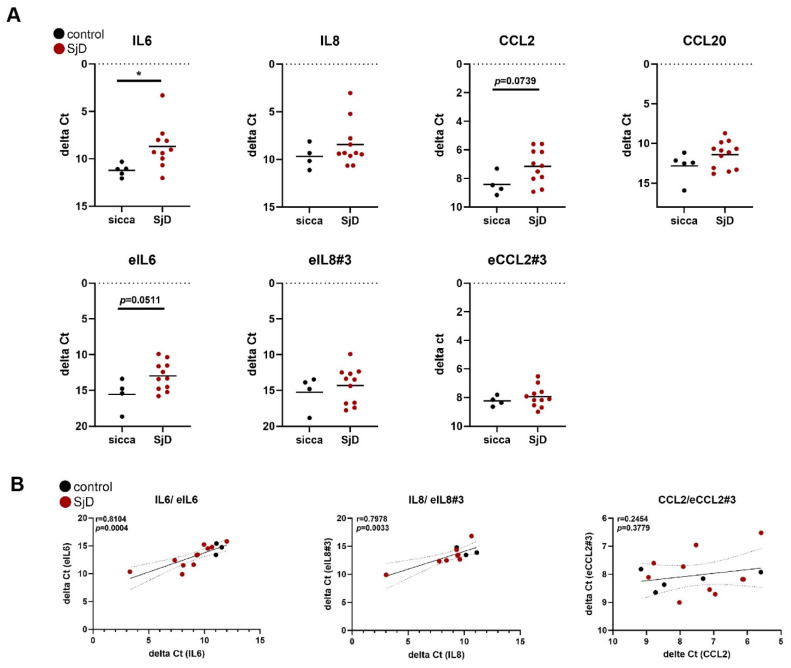
Expression of eRNAs in MSG tissues. (**A**) The expression of eRNAs and their corresponding coding transcripts in MSG tissues was measured by real-time PCR. (**B**) Correlation of eRNAs and coding transcripts in MSG tissues. Within the SjD group, patients with additional autoimmune diseases were shown as squares. * *p* < 0.05.

To verify whether eRNAs can be detected in patient tissue samples, we measured the expression of selected eRNAs and their corresponding coding genes in MSG biopsies of patients with SjD and controls. The expression of IL6 and eIL6 was increased in MSG tissues of patients with confirmed SjD compared to control tissues and positively correlated with each other (Figure 5A,B).

In addition, levels of IL8 and eIL8#3 positively correlated in MSG tissues, with no differences in their levels of expression between patients with SjD and controls. Whereas levels of CCL2 were increased in patients with SjD compared to controls, levels of eCCL2#3 were not different between the two groups and did not correlate with each other. This suggests that either other eRNAs for CCL2 are more important in regulating CCL2 expression in tissues, or the expression of eCCL2#3 is not sustained in absence of constant pro-inflammatory stimuli. The expression of eCCL20, in contrast to CCL20, was only detected in three out of 17 MSG tissues, in line with the short-term expression of this eRNA in stimulated SGF, with a peak at 1 h. We did not detect differences in samples of patients with SjD only and SjD patients with additional autoimmune diseases for all measured eRNAs and corresponding coding transcripts in salivary gland tissues.

### 3.5. Bromodomain Protein Inhibition Suppresses eRNA and Coding Gene Expression

**Figure 6:**
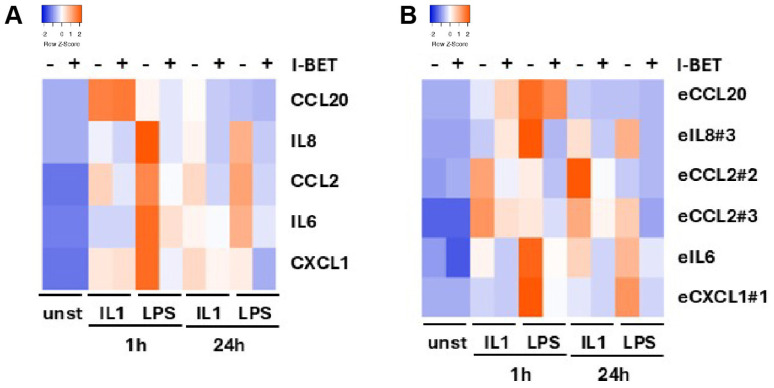
Anti-inflammatory effects of bromodomain protein inhibition in SGF. SGF were stimulated with IL1 or LPS for 1 h and 24 h in the absence and presence of I-BET151. The expression of (**A**) coding transcripts and (**B**) corresponding eRNAs was measured by real-time PCR. Individual data points for data presented in heatmaps are shown in Supplementary Figure S4.

To study the regulation of eRNA expression, we treated SGF with and without the pan-BRD protein inhibitor I-BET151. Consistent with the known anti-inflammatory effects of BRD protein inhibitors, I-BET151 suppressed the IL-1- and LPS-induced expression of pro-inflammatory cytokines and chemokines (Figure 6A, Supplementary Figure S4). In addition, I-BET151 suppressed the expression of the corresponding eRNAs in SGF at both 1 h and 24 h (Figure 6B, Supplementary Figure S4).

### 3.6. Silencing of eRNA Expression Suppresses Coding Gene Expression

**Figure 7:**
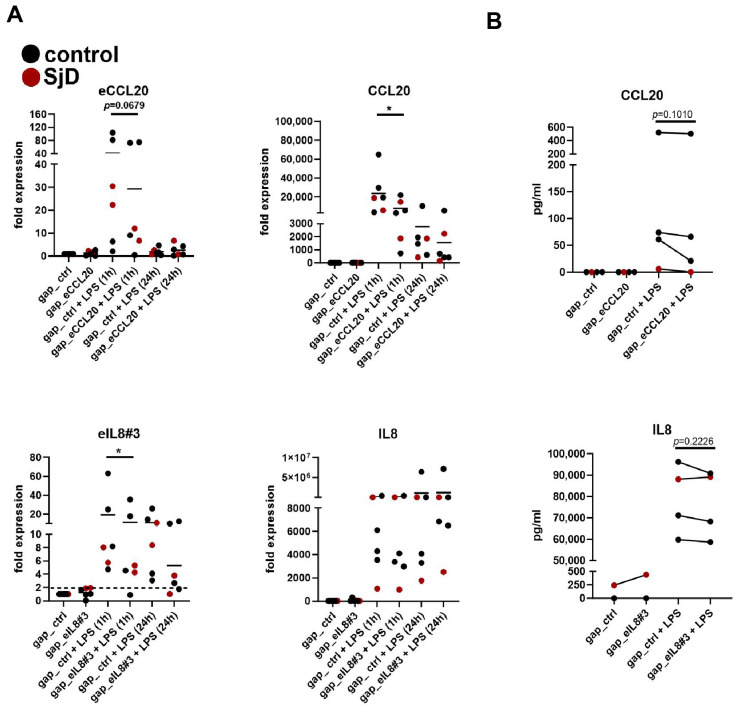
Modulation of eRNA expression in SGF. (**A**) SGF were transfected with gapmeR targeting eCCL20 (gap_eCCL20), gapmeR targeting eCCL8#3 (gap_eIL8#3), or a non-targeting control (gap_ctrl) prior to simulation with LPS for 1 h and 24 h. The mRNA expression of eRNAs, corresponding coding transcripts was measured by real-time PCR. (**B**) Protein secretion into cell culture supernatants was measured by ELISA. * *p* < 0.05.

To confirm the interaction of eRNAs with their coding genes, we have silenced selected eRNAs (eIL8#3, eCCL20) in SGF prior to stimulation with LPS. Transfection with gapmeR targeting eCCL20 prevented its LPS-induced expression by 28.7% (±84.4%) after 1 h of stimulation. At 24 h of stimulation, eCCL20 was not different between controls gap_eCCL20-transfected cells, explainable by the low expression levels of eCCL20 at this time point. In line with the reduced eCCL20 levels in gap_eCCL20-transfected cells, the mRNA expression and secretion of CCL20 were reduced (Figure 7A,B). This indicates that eCCL20 is crucial for regulating CCL20 expression. Transfection with gapmeR targeting eIL8#3 prevented its LPS-induced expression by 40.5% (±69%) after 1 h of stimulation and by 52.6% (±42.7%) after 24 h of stimulation. We detected only a minor decrease of LPS-induced IL8 expression at 1 h of stimulation, and a slight reduction of IL8 secretion (Figure 7A,B). Given that we have identified several eRNAs for IL8, reducing the expression of only one eRNA was not sufficient to sustainably affect levels of IL8.

## 4. Discussion

Salivary dysfunction is a hallmark of SjD. Studies in primary cultures of SGEC from salivary gland tissues have provided new insights into their role in B cell survival and B and T cell activation [23,24]. In contrast to SGEC and immune cells, fibroblasts represent an understudied cell type in SjD. We confirmed the stromal origin of cultured SGF by FACS analysis. In this study, we provide the first data sets on the activation and pro-inflammatory response of cultured SGF from patients with confirmed SjD and controls not fulfilling the classification criteria. There is increasing evidence that SGF potentially get activated and play an early role in SjD. In mouse models, in which salivary gland inflammation was mimicked by viral application into salivary glands, SGF got activated and expanded before immune cell infiltration [8]. Furthermore, fibroblast activation is a key event in the formation of tertiary lymphoid structures (TLS) in inflamed salivary glands [8,25], which are associated with severe disease [26].

We detected several cytokines and chemokines induced by different pro-inflammatory stimuli in SGF, indicating that these cells are equipped to serve as amplifiers of an inflammatory response once activated. With the activation of VCAM1 and ICAM1, cultured SGF expressed two adhesion molecules that characterize activated immunofibroblasts which were described in mouse models of salivary gland inflammation and in inflamed human salivary gland tissues of patients with SjD [8,25]. Furthermore, CXCL10 has been identified, along with CCL19, as a marker for immune cell-interacting fibroblasts in chronically inflamed salivary glands [10]. Here, we show that SGF induce a profound pro-inflammatory response upon stimulation with IL1 and pIC, and activate with “NF-kappa B signaling”, “JAK STAT signaling”, and “interferon signaling” pathways, which are of high relevance in SjD [4,27]. Given that inhibitors targeting “JAK STAT” signaling, as well as anti-BAFF, are currently tested in clinical trials in SjD [4], such inhibitors might therefore have effects beyond immune and epithelial cell compartments by targeting additional fibroblasts.

The pro-inflammatory response in vitro was similar in SGF from patients with SjD and controls not fulfilling the classification criteria, at least for the measured target genes. However, we detected differences in proliferation between cultured SGF from patients with SjD and controls, with cells from patients with SjD exhibiting increased proliferation rates. This may indicate that the microenvironment within MSG tissues of SjD patients induces an activation state that is maintained in cell culture.

Fibroblast activation is a characteristic of chronically inflamed tissues, as demonstrated by Korsunsky et al. in inflamed tissues from the joint, salivary gland, lung, and gut. In chronic inflammation, shared fibroblast subtypes support tissue reorganization and define perivascular and lymphocyte-enriched niches [10]. Given the similarities between fibroblasts from different tissues [10], we compared their activation under pro-inflammatory conditions by analyzing the transcription of eRNAs, a hallmark of active enhancers [14]. Enhancers significantly contribute to the development of autoimmune diseases as they are enriched in genetic risk variants and alter cellular responses in a cell type- and stimulus-specific manner [28]. In addition to immune cells, fibroblasts contribute to disease heritability, as shown for RA, where FLS account for up to 24% of heritability [19]. The measurement of eRNAs provides a straightforward method for detection of active enhancers. Given our available CAGE-seq data sets of FLS [19], we were able to extract eRNA sequences and to measure eRNA expression cost-effectively in a time-resolved manner upon stimulation with different pro-inflammatory stimuli by using real-time PCR. We identified several eRNAs for pro-inflammatory cytokines and chemokines that are shared among fibroblasts from different tissues. The expression of some eRNAs was maintained for up to 24 h, contradicting previous reports that eRNAs are short-lived [14]. Given that we detected eRNA expression not only in cultured fibroblasts but also in MSG tissue samples, our findings have in vivo relevance.

In a proof-of-concept experiment using the bromodomain inhibitor I-BET, we were able to demonstrate the possibility to interfere with pro-inflammatory enhancer activation in SGF. BRD inhibitors, such as JQ-1 and I-BET, showed strong anti-inflammatory effects in several rheumatic and musculoskeletal diseases across different cell types, including FLS [29]. With this study, we provide the first evidence of the potential anti-inflammatory efficacy of BRD protein inhibitors in SjD. I-BET treatment of FLS was shown to globally decrease levels of acetylated histones, both in unstimulated and TNF-stimulated FLS [30]. Active enhancers are marked by histone acetylation. In line with this, the BET inhibitor JQ1 was reported to antagonize not only the synthesis and elongation of protein-coding transcripts but also that of non-coding eRNA transcripts in a bromodomain-dependent manner [31].

## 5. Conclusions

SGF, similar to FLS and LF, respond to different pro-inflammatory stimuli by inducing the expression of transcribed eRNAs and their corresponding coding genes. BRD inhibitors are sufficient to prevent the activation of eRNAs and pro-inflammatory cytokine and chemokine expression. Local SGF activity in SjD may promote the vicious cycle of chronic inflammation in salivary glands.

## Figures and Tables

**Table 1:** Clinical characteristics of patients with SjD or sicca syndrome.

Diagnosis	SjD (*n* = 20)	Sicca (*n* = 15)	*p* Value
age (years, mean [range])	53.2 (26–83)	55.2 (37–74)	0.6638
sex (male/female)	3/17	4/11	0.4081
other autoimmune disease (yes/no)	5/15	2/13	0.6722
Chisholm-Mason grade (range)	3.1 (1–4)	1.27 (0–2)	0.0001
focus score (range)	1.81 (0–5)	0.16 (0–0.8)	0.0001
anti-SSA/Ro positive (yes/no)	13/7	1/14	0.0002
Schirmer test pathological (yes/no/ND)	(14/3 */3 **)	(8/1/6 **)	0.7558
UWS flow pathological (yes/no/ND)	(3/0/17)	(0/0/15)	-
CRP (mg/L range; ND)	4.3 (0–32; 4)	1.6 (0.6–5.4; 6)	0.4308
IgG (g/L range; ND)	10.4 (4.9–17; 12)	9.18 (7–12; 10)	0.4784
treatment	eye drops (2) DMARD (6)	DMARD (1)	-
extra-glandular symptoms	articular (4) Raynaud’s phenomenon (3) renal (1) small fiber neuropathy (1) central nervous system (1) cutaneous (1) pulmonary (1) myocardial fibrosis (1)	polyneuropathy (1)	-

CRP, C reactive protein; DMARD, Disease-Modifying Antirheumatic Drugs; ND not determined; IgG, immunoglobulin G; UWS, unstimulated whole saliva; * patients had a pathological sialometry; ** patients that were referred by external physicians for a biopsy.

**Table 2:** Location of eRNAs associated with cytokines and chemokines.

Coding Gene	eRNA	Location Relative to TSS	Distance to TSS	Location in hg19
*IL6*	eIL6	upstream	0.5 kb	chr7:22766294-22766470
*IL8*	eIL8#1	upstream	14.1 kb	chr4:74591586-74592042
	eIL8#2	upstream	4.7 kb	chr4:74600999-74601413
	eIL8#3	upstream	18.1 kb	chr4:74587655-74588079
	eIL8#4	upstream	35.6 kb	chr4:74570129-74570560
*CCL2*	eCCL2#1	upstream	3.1 kb	chr17:32579403-32579877
	eCCL2#2	upstream	0.3 kb	chr17:32580999-32581402
	eCCL2#3	downstream	1.4 kb	chr17:32584373-32584831
	eCCL2#4	downstream	3.2 kb	chr17:32586187-32586639
*CCL20*	eCCL20	intronic	0.3 kb	chr2:228678859-228679309
*CXCL1*	eCXCL1#1	downstream	47.8 kb	chr4:74782759-74783271
	eCXCL1#2	upstream	31.5 kb	chr4:74702960-74703446

eRNA: enhancer RNA; TSS: transcription start site; kb: kilo base.

## Data Availability

Data are contained within the article.

## Supplementary Materials

The following supporting information can be downloaded at: https://www.mdpi.com/article/10.3390/cells14080558/s1, Figure S1: FACS analysis of SGF; Figure S2: Interaction of eRNAs with coding genes; Figure S3: Different pro-inflammatory stimuli induce the expression of eRNAs in FLS and LF; Figure S4: I-BET has anti-inflammatory effects in SGF; Table S1: Clinical characteristics of patients with RA used for cultivation of synovial fibroblasts (FLS); Table S2: Sequences of primers used for real-time PCR; Table S3: Pathway enrichment analysis of SGF stimulated with IL1; Table S4: Pathway enrichment analysis of SGF stimulated with pIC.
