# Supplementary material for: Pro-Inflammatory Properties of Salivary Gland-Derived Fibroblasts—Implications in Sjögren’s Disease
Source: Cells. 2025 Apr 8;14(8):558. doi: 10.3390/cells14080558 (PMC12025495; doi:10.3390/cells14080558)
Supplement: Supplementary file 1 [file cells-14-00558-s001.zip › supplementary figures.pdf]

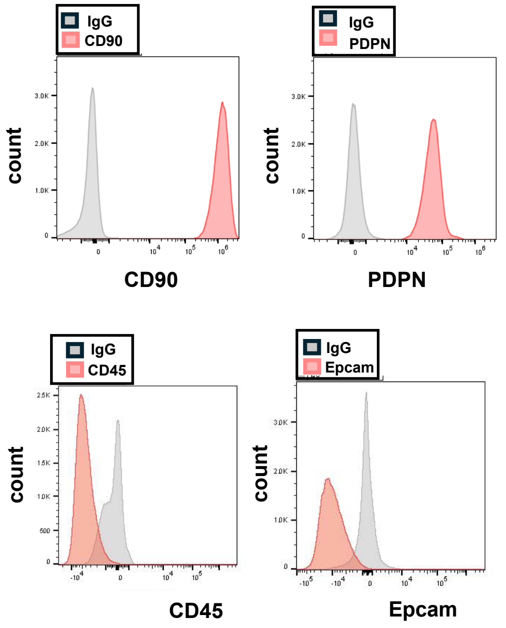

Supplementary Figure S1: FACS analysis of SGF

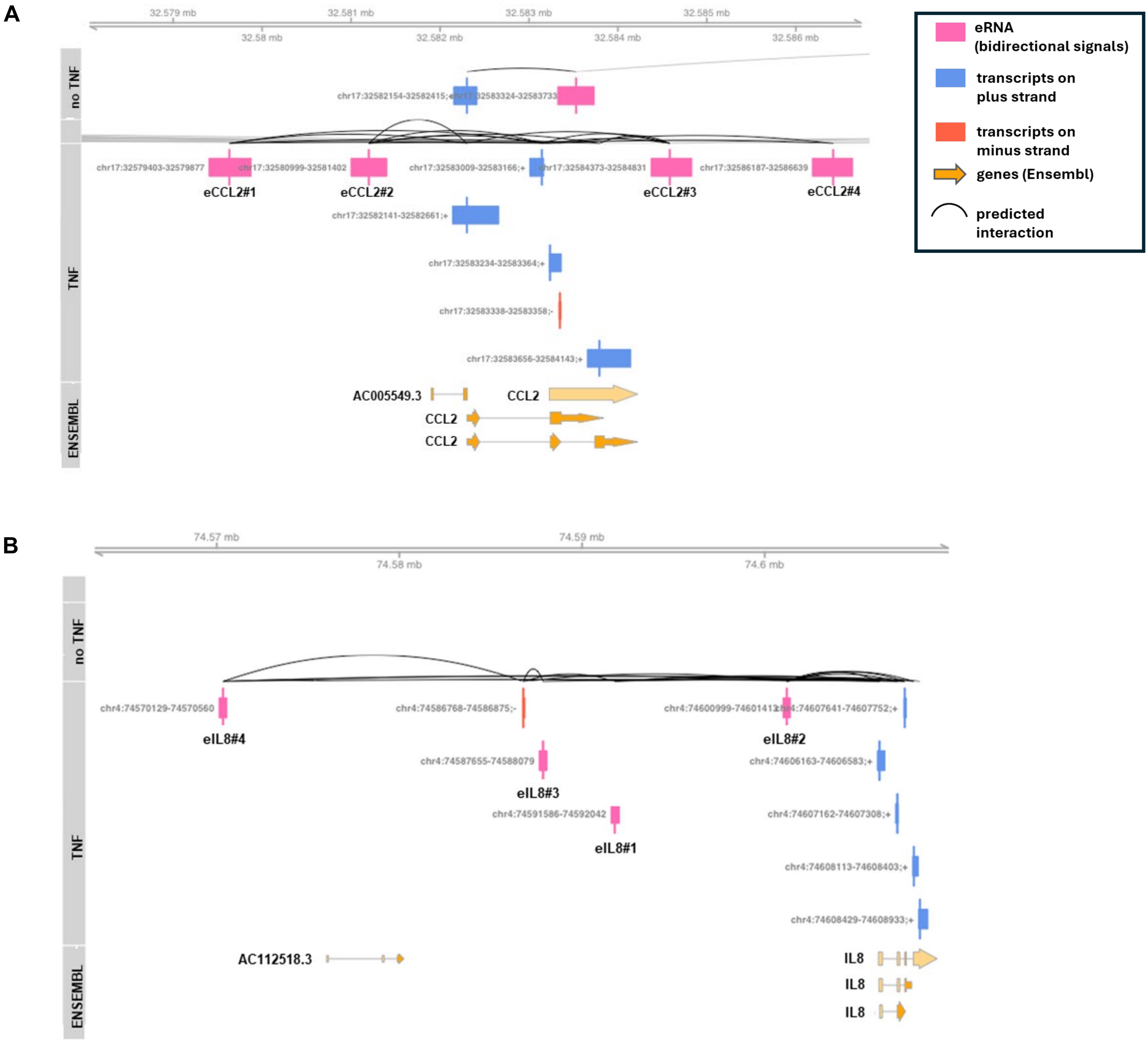

Supplementary Figure S2: Interaction of eRNAs with coding genes. The expression of bi-directionally transcribed eRNAs for **A.** CCL2 and **B.** IL8 in unstimulated and TNF-stimulated FLS was identified by CAGE-sequencing.

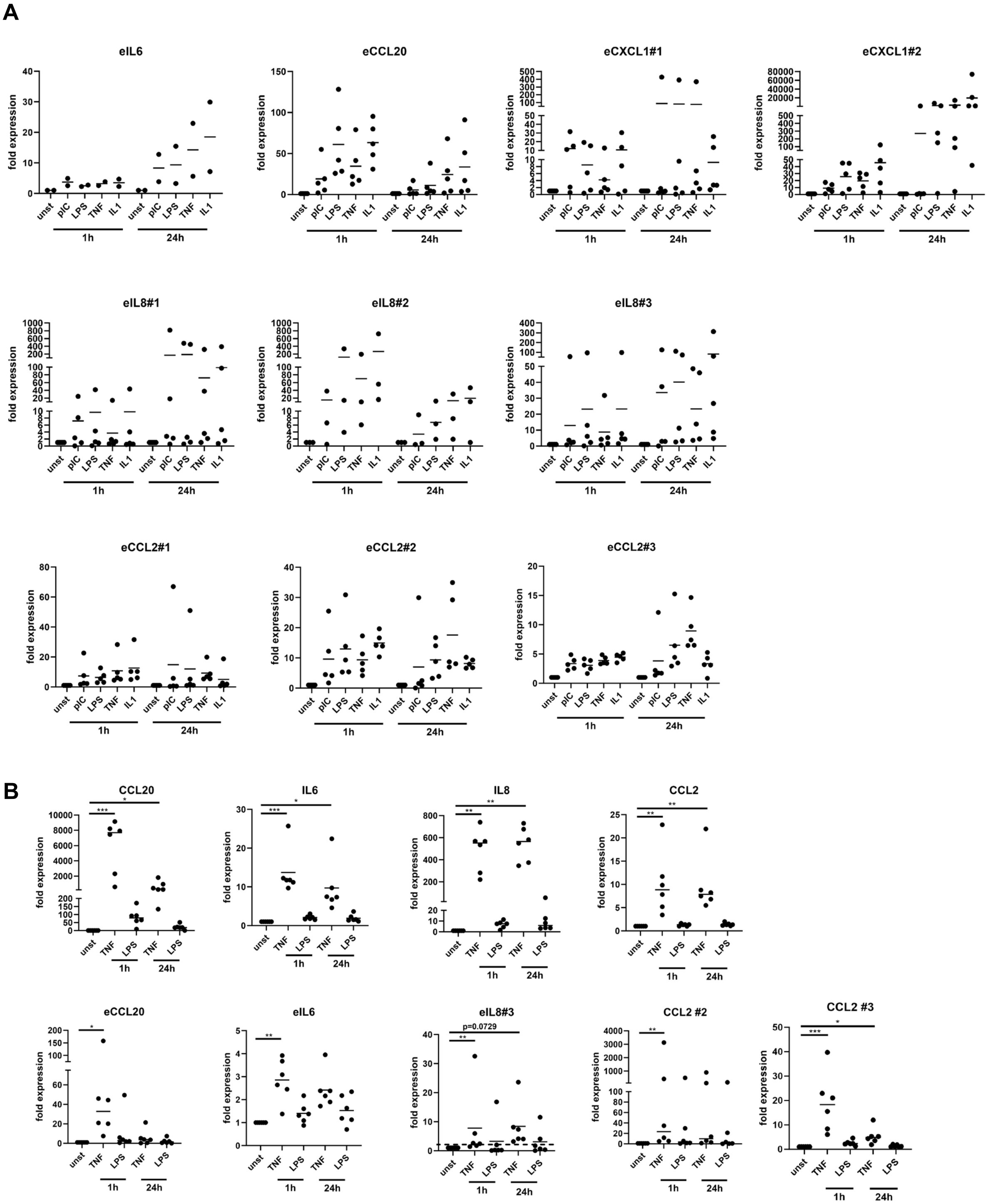

Supplementary Figure S3: Different pro-inflammatory stimuli induce the expression of eRNAs in FLS and LF. **A.** FLS were stimulated with pIC, LPS, TNF and IL1 for 1 and 24 hours. **B.** LF were stimulated with TNF or LPS for 1 and 24 hours. The expression of eRNAs was measured by Real-time PCR.

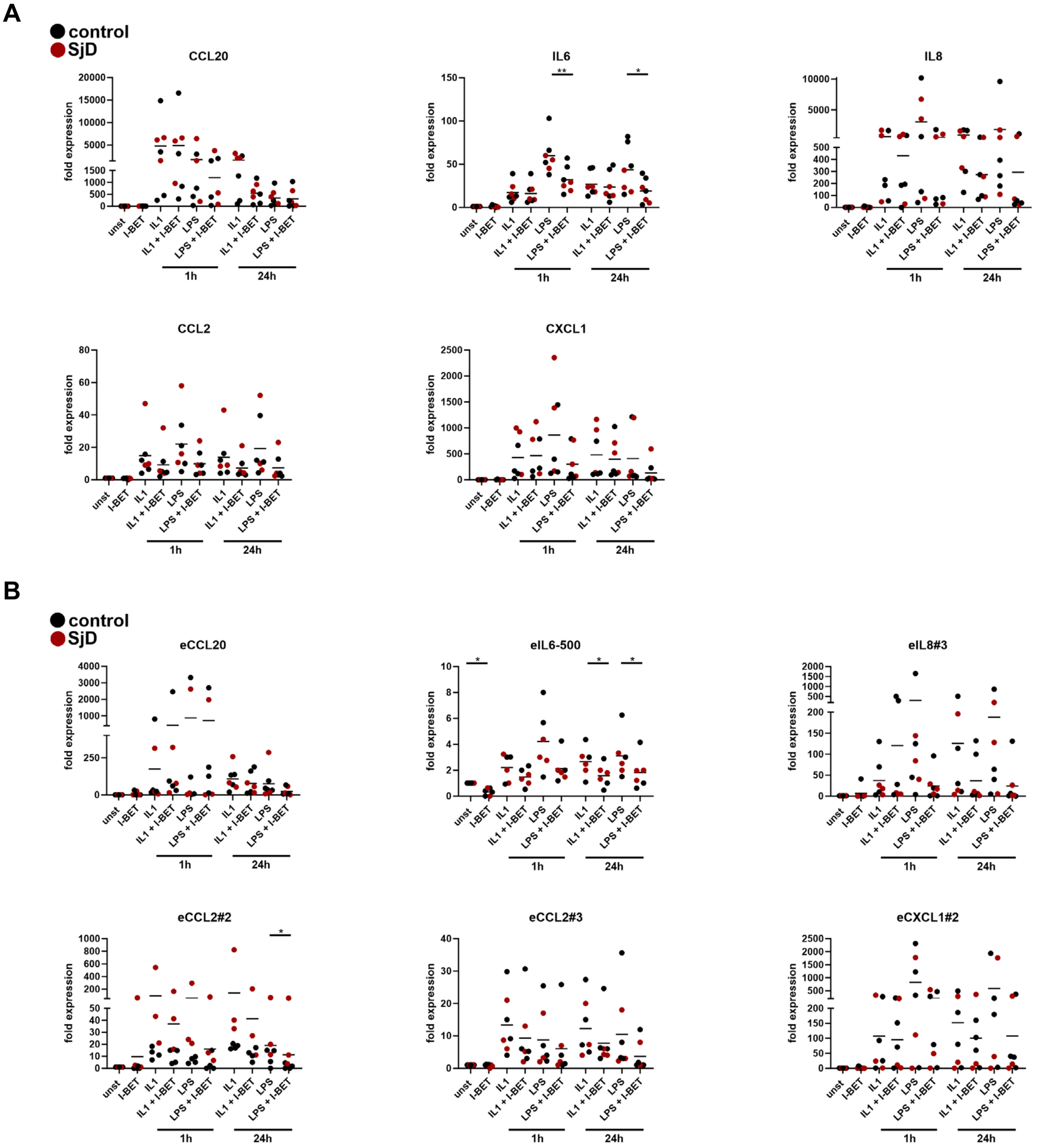

Supplementary Figure S4: I-BET has anti-inflammatory effects in SGF. SGF were stimulated with IL1 or LPS for 1 and 24 hours, or DMSO (unstimulated). The expression of **A.** coding transcripts and **B.** eRNAs was measured by Real-time PCR.
