# Supplementary material for: Pro-Inflammatory Properties of Salivary Gland-Derived Fibroblasts—Implications in Sjögren’s Disease
Source: Cells. 2025 Apr 8;14(8):558. doi: 10.3390/cells14080558 (PMC12025495; doi:10.3390/cells14080558)
Supplement: Supplementary file 1 [file cells-14-00558-s001.zip › Supplementary Table S1.pdf]

Supplementary Table S1. Clinical characteristics of patients with RA used for cultivation of synovial fibroblasts (FLS)

Diagnosis	RA(18)/ Sicca(1)
age (years, mean (range))	43, (20-62)
sex (male/female)	6/ 13
Joint	Finger (4)<br>Knee (2)<br>Shoulder (7)<br>Thumb (2)<br>Wrist (4)
CRP (mg/l)	15.3(0.5-62.1); 6 ND
Rheumatoid factor (positive/negative/ ND)	14/1/4
Treatment	Glucocorticoids (4)<br>DMARD (8)<br>Biologics (4)<br>None (1)<br>ND (5)

CRP, C reactive protein; DMARD, Disease-Modifying Antirheumatic Drugs; ND, not determined; RA, rheumatoid arthritis.
