# Supplementary material for: Pro-Inflammatory Properties of Salivary Gland-Derived Fibroblasts—Implications in Sjögren’s Disease
Source: Cells. 2025 Apr 8;14(8):558. doi: 10.3390/cells14080558 (PMC12025495; doi:10.3390/cells14080558)
Supplement: Supplementary file 1 [file cells-14-00558-s001.zip › Supplementary Table S2.pdf]

**Primers for coding transcripts**	Sequence (5`- 3`)
RPLP0 fw	GCG TCC TCG TGG AAG TGA CAT CG
RPLP0 rev	TCA GGG ATT GCC ACG CAG GG
IL6 fw	CCC TGA GAA AGG AGA CAT GTA AC
IL6 fw	CCT CTT TGC TGC TTT CAC ACA TG
IL8 fw	TTG GCA GCC TTC CTG ATT TC
IL8 fw	TGG CAA AAC TGC ACC TTC AC
BAFF fw	AGG GTC CAG AAG AAA CAG TCA
BAFF rev	TGT ATA GTT GGT GTT TCA CTG TCT G
CCL20 fw	GAC ATC AAT GCT ATC ATC TTT CAC AC
CCL20 rev	TGG ATT TGC GCA CAC AGA CAA C
CCL2 fw	CTC GCT CAG CCA GAT GCA ATC
CCL2 rev	AAG TTA TAA CAG CAG GTG ACT GG
CXCL1 fw	GAG AGA CAC AGC TGC AGA GGC
CXCL1 rev	TGC TCA AAC ACA TTA GGC GC
ICAM1 fw	CGT GAA TGT GCT CTC CCC C
ICAM1 rev	AGT GCC CAT TAT GAC TGC GG
VCAM1 fw	GTA AAA GAA TTG CAA GTC TAC ATA TCA
VCAM1 rev	GAT GGA TTC ACA GAA ATA ACT GTA TTC
**Primers for eRNAs**	
eCCL20 fw	CAG ATT TGG GGA GTG GTG GGC
eCCL20 rev	ATC ACT CCC AGA AAC ATC ACT TTG GC
eIL6 fw	GGA ACG CTA AAT TCT AGC CT
eIL6 rev	AGT CAT GCA CGA AGT TTT AC
eIL8#1 fw	CAC ATG GTC AAG GGG CTC ATA CC
eIL8#1 rev	ATA AAA TCC TGT GCA ATG TCT TTG GCT GAC
eIL8#2 fw	CAT CAG TTC AGT CAT TTC TAA CAT AGC AAC
eIL8#2 rev	CAC TTA GGC ATT ATT TAC TCC AAT GTT TAC
eIL8#3 fw	CAC TCC AGG GCT GGC CTT ATA AG
eIL8#3 rev	GAC AAG GGG CAT TCC CAG ATC TC
eCCL2#1 fw	TTT GTG CCA GAG CCT AAC C
eCCL2#1 rev	AGT TCC CAG ATC CCG TAG AA
eCCL2#2 fw	GAA GTC AGG CTT TCC AAT TCC CG
eCCL2#2 rev	GCA GTG GAC AAG AAG TTC ATC CC
eCCL2#3 fw	GTC ACC CAT TTA GCA AGA GCC C
eCCL2#3 rev	CAT GCA GGG GTC AGG GTC TG
eCXCL1#1 fw	GAC ACC ACC ATA ATT CTG AGG TAT G
eCXCL1#1 rev	CAG AGA CTA TGA GGG AAT CAG GTC
eCXCL1#2 fw	CAC AAT GTC CTT ATT CTC TCT GTA GG
eCXCL1#2 rev	GGT TTA CCC AGA ACA TTC CAG AAA AAA AG

Supplementary Table S2: Sequences of primers used for Real-time PCR
